# Determination of online thin film buckling configuration by parametric optimization for flexible sensor application

**DOI:** 10.1038/s41598-023-37666-0

**Published:** 2023-07-04

**Authors:** Yeoun-Jae Kim, Daehan Wi, Jingyu Kim, Jaesoon Choi

**Affiliations:** 1 Biomedical Engineering Research Center, Asan Institute for Life Sciences, Asan Medical Center, 88, Olympic-ro 43-gil, Songpa-gu, Seoul, 05505 South Korea; 2 Department of Biomedical Engineering, Asan Medical Center, University of Ulsan College of Medicine, Seoul, 05505 South Korea

**Keywords:** Biomedical engineering, Sensors and biosensors, Mechanical engineering

## Abstract

A mini basket type mapping catheter consists of thin film flexible sensors and is applied in the medical field to measure the electrocardiography (ECG) signals in order to localize and quantize the physiological condition/status of heart. The flexible nature of the thin film changes the configuration with respect to the contact boundary conditions when it contacts a target surface. Therefore, to accurately localize the flexible sensor, the thin film flexible sensor’s configuration must be determined accurately in an on-line fashion. As a study of localizing the thin film flexible sensor, this study proposes an on-line thin film buckling configuration determination method using parametric optimization and interpolation technique. With the specific modulus of elasticity and dimensions of the thin film flexible sensor of the mapping catheter prototype, the buckling configuration with two point boundary condition under axial load can be calculated in desktop environment. The proposed calculation method is validated by mapping catheter sensor prototype test. The calculation/test results showed that the maximum overall length L, *x*$_{a}$, and *y*$_{a}$ value error between the calculation and experiment are approximately 0.16 mm, − 0.12 mm. − 0.10 mm in 50 ms calculation time. The calculation result of the proposed method is also compared with that of the numerical simulation by FEM, which has approximately 0.44 mm *y*$_{a}$ value error compared with that of the experiment.

## Introduction

Thin films made of polyethylene terephthalate (PET), polystyrene (PS), and cellulose nanofibers (CFG) can be used as flexible sensors when manufacturing through certain processes by embedding microelectrons between the films^1,2^. Because the thin film has asymmetric flexural rigidity (EI), it buckles when axial force is applied with various boundary conditions and restores its configuration when the axial force is released. These buckling/restoring characteristics make the flexible sensor a good candidate for medical usage of ECG signal measurement, which includes inserting the sensor in the right atrium while guiding it through right aorta with minimal volume and buckling it to localize and measure the ECG signals inside the right atrium^3,4^. The flexible sensors are called mapping catheters, and various types of them are used in the medical field^3,4^. Especially, mini-basket shaped high resolution mapping catheter^5–7^ features more clarity for precise localization.

**Figure 1:**
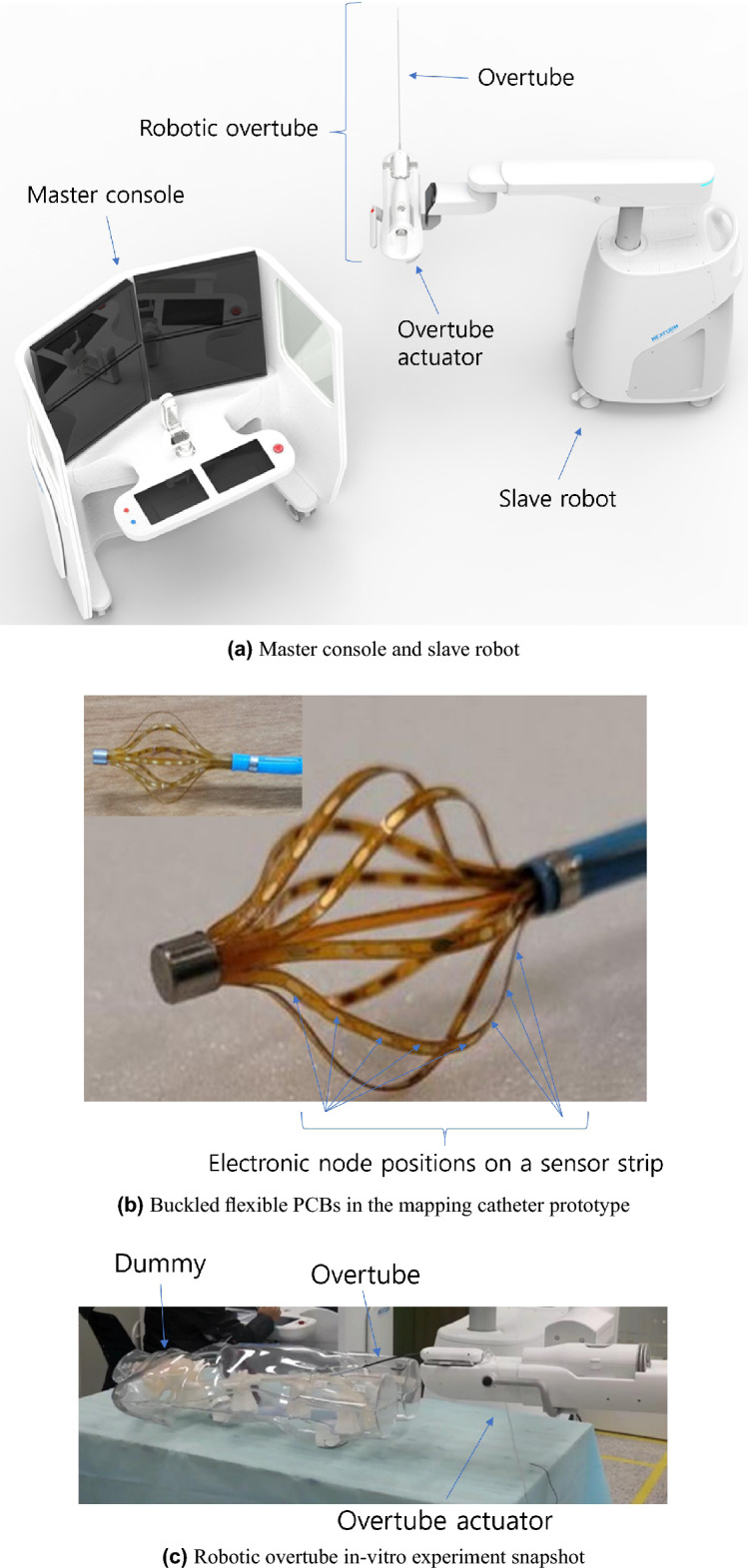
The master-slave robot system and mapping catheter prototype developed for the heart arrhythmia surgery.

The authors have been involved in developing an intelligent cardiovascular intervention assist robot with cardiac mapping system as shown in Fig. 1a, which describes a master console and slave robot. An in-vitro experiment with the robotic overtube, which is installed in the end-effector of the slave robot, with three degree of freedom (bending, yawing, translating) is presented in Fig. 1c. Accompanied with the developed robot system, a mini basket type mapping catheter prototype is also developed as shown in Fig. 1b, to be inserted coaxially within the overtube and used for performing remote and accurate cardiac arrhythmia mapping while manipulating the robotic overtube with master system. The prototype consists of eight flexible printed circuit boards (PCB) that could adjust axial distance between the two boundaries, and it is similar to the commercialized high-resolution mini-basket mapping catheter used in the Rhythmia mapping system^5,8^. It can be manipulated manually or by cardiovascular intervention assist robot.

However, in order to correctly localize the ECG signal with the mapping catheter prototype, it is important to accurately calculate the buckled configuration of the flexible PCB in the prototype with respect to an adjusted axial distance. The column buckling, including thin film buckling, have been extensively studied worldwide, which includes exact solution by elliptic integral^9–15^, exact solution approximation by perturbation theory^16,17^, and FEM(Finite Element Method) by numerical integration^18–24^.

Timoshenko et al.^9^ investigated and summarized the classical solution for buckling of elastic bar with an axial load in clamped boundary. They analytically induced the lateral deformation and critical load with respect to flexural rigidity by implementing the change of variables and elliptic integral. Hubbard^10,11^ introduced an iterative numerical solution for pole vault problem, which is a large deformation of buckled bar under axial and lateral load in clamped boundary. Griner^12^ induced a parametric solution to the pole-vault problem, wherein the tabulated elliptic integral is used. There are exact solutions for other boundary conditions. Panayotounakos et al.^13^ induced closed form solution for the elastica of straight bars under uniform distributive forces. Mikata^14^ induced an exact post-buckling solution of elastica for a clamped-hinged beam with its application to a carbon nanotube. Armanini et al.^15^ integrated Euler’s elastica to determine the configuration of elastica compass and catapult, in which the load is applied at one side and the load is slowly rotated. They compared the exact solution with finite-element scheme.

The exact solutions of Elastica include elliptic integral of 1st and 2nd kind. To calculate the actual configuration, they must be numerically integrated^25,26^. Berkey^16^ performed an asymptotic analysis of elastic column, pinned at both ends and subjected to an axial thrust, with perturbation method. Wang^17^ suggested a buckling configuration approximation by perturbation theory to approximate the configuration of inclined cantilever beam with vertical end load. The aforementioned exact solutions and analytical approximations are rather classic and depends on mathematical rigor without any needs for large computing capability. However, FEM-based method is quite well established with commercialized software^18–21^ and opensource software^22–24^ with generalized purposes, such as structure with composite materials and complex geometry.

The mapping catheter prototype in Fig. 1b has composite structure and layers, which make it hard to apply the classical exact solutions and analytic approximations directly to the accurate configuration determination of this model. FEM based methods could be ideal for solving the buckling configuration of thin film with composite structure like this model. However, the commercial FEM software are usually expensive, and open source software generally needs additional accuracy validation. Moreover, because the applied forces and moments are not known beforehand in calculating the configuration, inverse problem must be solved, and the forces and moments also must be calculated with respect to the geometric constraints and boundary conditions. Since the flexible PCB in the mapping catheter prototype is in a free-standing column buckling (FSCB) state when an axial load is applied, the compressive axial force *P* and maximum bending angle $\alpha$ are required to be determined, which is not feasible for the flexible PCB in the mapping catheter prototype because only the axial distance between the fixed boundaries are known.

Therefore, to solve these kinds of problems, a parametric optimization framework, accompanied with classical exact solution, is proposed in this study. The proposed method uses several analytical solutions by Elastica and gradient-based optimization algorithm. Not only does it take less time to calculate, but it can also be done online, which are outstanding advantages over FEM based method with large number of mesh points and inverse matrix calculation. Moreover, even though the isotropic material assumption in the exact solution is not accurate in the mapping catheter prototype with embedded electronic circuit in Fig. 1b, the error can be absorbed by parametric optimization process in finding the axial force *P* and maximum bending angle $\alpha$ which best fit for the measured boundary conditions and geometric constraints. In this framework, a cheaper and accurate configuration determination can be possible. The contribution of the work are as follows.

1. A method for determining accurate and inexpensive thin film buckling configuration in clamped boundary using a parametric optimization and classical exact solution solver is proposed. This method can be applied to any composite structured thin film through the optimization process, and the lumped material assumption error can be offset in the inverse problem solving by the parametric optimization.
2. For the proposed parametric optimization, the steepest descent method is implemented first. An optimization variable space based divide and conquer method is proposed to solve the hunting phenomenon occurred during optimization process and to get accurate solution.
3. The feasibility of the proposed method is verified by comparing the calculation results with those of FEM based method using ANSYS software^18^ and experiments with a screen protector strip and the mapping catheter prototype.

The contents are organized as follows. In “Thin film buckling configuration determination method” Section, the problem definition and the proposed method with temporary PET film result is presented. In “Proposed method (algorithm 1), measurement, and fem simulation results” Section, the measurements, FEM simulations^18^, and the results of the proposed method are compared with the flexible PCB sensor in the mapping catheter prototype. The conclusions and future works are presented in “Conclusion” Section.

## Thin film buckling configuration determination method

### Preliminaries and classical Solution

**Figure 2:**
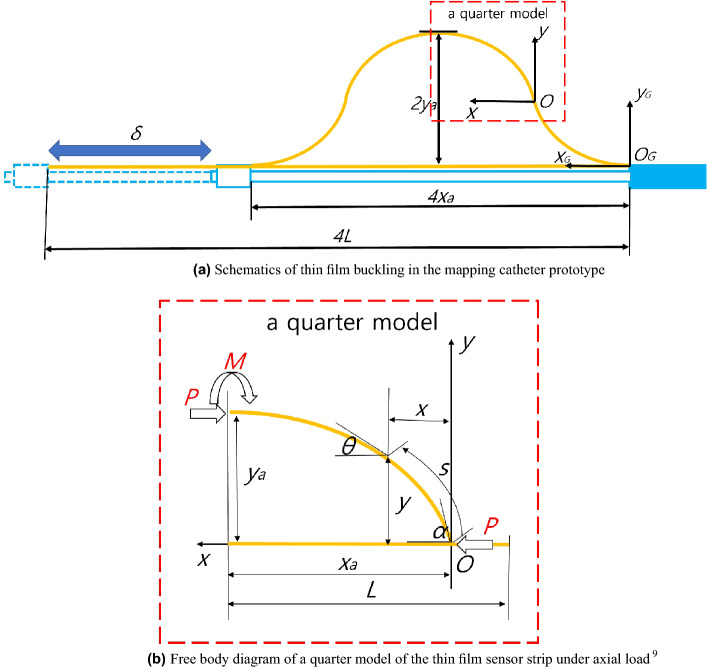
Schematics and free body diagram of a thin film strip sensor buckling in the mapping catheter prototype.

The schematics and free body diagram of thin film buckling in the mapping catheter prototype are presented in Fig. 2a,b, respectively.

Out of 8 buckled yellow film sensors in the mapping catheter prototype in Fig. 1b, a planar schematic of one yellow film sensor is presented in 2(a).

In Fig. 2a, the mapping catheter moving rod is represented in sky blue, which is assembled to the main body in the rightmost side of Fig. 2a and fixed horizontally. A thin film strip in Fig. 2a is represented in yellow, which is fixed at both ends of the moving body and its total length is 4*L*. If the length of the moving rod is shortened to $\delta$, the configuration of thin film strip changes from a straight line to a buckled shape, with a 4*x*$_{a}$ width and a 2*y*$_{a}$ height, respectively. The two coordinate systems in Fig. 2a represents the global coordinates ($O_{G}-x_{G}-y_{G}$) and local coordinates $(O-x-y)$. The global coordinates system represents the base coordinates of the whole configuration of the thin film strip sensor, whereas the local coordinates system is for a quarter model of the thin film strip sensor, which is depicted in the red dotted rectangle in Fig. 2a. If the configuration of the quarter model is determined, the overall configuration in Fig. 2a can be determined by making a symmetric mirror image with respect to point O from the quarter model and then mirroring the half model with respect to $y_{G}$ axis at ($2x_{a}$, 0) position, which is explained in^9^.

The free body diagram of the quarter model of the thin film sensor strip is shown in Fig. 2b. A magnified view of the quarter model of thin film sensor in Fig. 2a is shown in Fig. 2b. The configuration path coordinates, *s* is also presented in Fig. 2b. The *x* and *y* values at *s* are also shown in Fig. 2b. The bending angle at the base is denoted by $\alpha$ and bending angle at s is denoted by $\theta$. The overall length of a quarter model in Fig. 2b is *L* and its width and height are $x_{a}$ and $y_{a}$, respectively. The compressive force is *P* and the bending momentum at *s = L* is *M* in Fig. 2b. Point *O* in Fig. 2a,b is the inflection point. The moment *M* in Fig. 2b is equal to $-Py_{a}$ by static equilibrium at *s = L*. The independent variables in Fig. 2b are $\alpha$ and *P* with fixed flexural rigidity *EI*. $s, x, y, x_{a}, y_{a}$ and *L* in Fig. 2b are analytically determined by Timoshenko et al.^9^ and repeated in Eqs. (1–9) for completeness of presentation.

1 $$\begin{aligned}{} & {} k = \sqrt{\frac{P}{{EI}}} \end{aligned}$$

2 $$\begin{aligned}{} & {} p = \sin \frac{\alpha }{2} \end{aligned}$$

3 $$\begin{aligned}{} & {} \sin \frac{\theta }{2} = p\sin \phi \end{aligned}$$

4 $$\begin{aligned}{} & {} L = \frac{1}{k}\int _0^{\frac{\pi }{2}} {\frac{{d\phi }}{{\sqrt{1 - {p^2}{{\sin }^2}\phi } }}} = \frac{1}{k}K(p) \end{aligned}$$

5 $$\begin{aligned}{} & {} {x_a} = \frac{2}{k}\int _0^{\frac{\pi }{2}} {\sqrt{1 - {p^2}{{\sin }^2}\phi } } d\phi - L = \frac{2}{k}E(p) - L \end{aligned}$$

6 $$\begin{aligned}{} & {} {y_a} = \frac{{2p}}{k}\int _0^{\frac{\pi }{2}} {\sin } \phi d\phi = \frac{{2p}}{k} \end{aligned}$$

7 $$\begin{aligned}{} & {} s = {\frac{1}{k}\int _0^{\frac{\pi }{2}} {\frac{{d\phi }}{{\sqrt{1 - {p^2}{{\sin }^2}\phi } }} - } \frac{1}{k}\int _0^{\phi } {\frac{{d\phi }}{{\sqrt{1 - {p^2}{{\sin }^2}\phi } }}}} \nonumber \\{} & {} \quad = \displaystyle {\frac{1}{k}K(p) - \frac{1}{k}\int _0^{\phi } {\frac{{d\phi }}{{\sqrt{1 - {p^2}{{\sin }^2}\phi } }}} } \end{aligned}$$

8 $$\begin{aligned}{} & {} x = \displaystyle {\frac{2}{k}E(p) - \frac{1}{k}K(p)}\nonumber \\{} & {}\,\,\,\,\,\,\,\,\,\,- (\frac{2}{k}\int \limits _0^{\phi } {\sqrt{1 - {p^2}{{\sin }^2}\phi } d\phi - } \frac{1}{k}\int \limits _0^{\phi } {\frac{{d\phi }}{{\sqrt{1 - {p^2}{{\sin }^2}\phi } }}} ) \end{aligned}$$

9 $$\begin{aligned}{} & {} y = \frac{{2p}}{k}\cos \phi \end{aligned}$$

*k* and *p* in Eqs. (1) and (2) are the independent variables coming from *P* and $\alpha$. *EI* is the flexural rigidity of the thin film with respect to the z axis in Fig. 2b. The configuration (x,y) is a function of s or $\theta$. $\theta$ is changed to $\phi$ by Eq. (3). With $\phi$ variable, the integral along $\theta$ = $\alpha$ to 0 can be changed to the integral along $\phi$ = 0 to 90$^\circ$. *K(p)* and *E(p)* in Eqs. (4, 5) and (7, 8) are complete elliptic integral of first kind and second kind, respectively.

### Problem definition and overall sequence of the determination of a thin film buckling configuration

**Figure 3:**
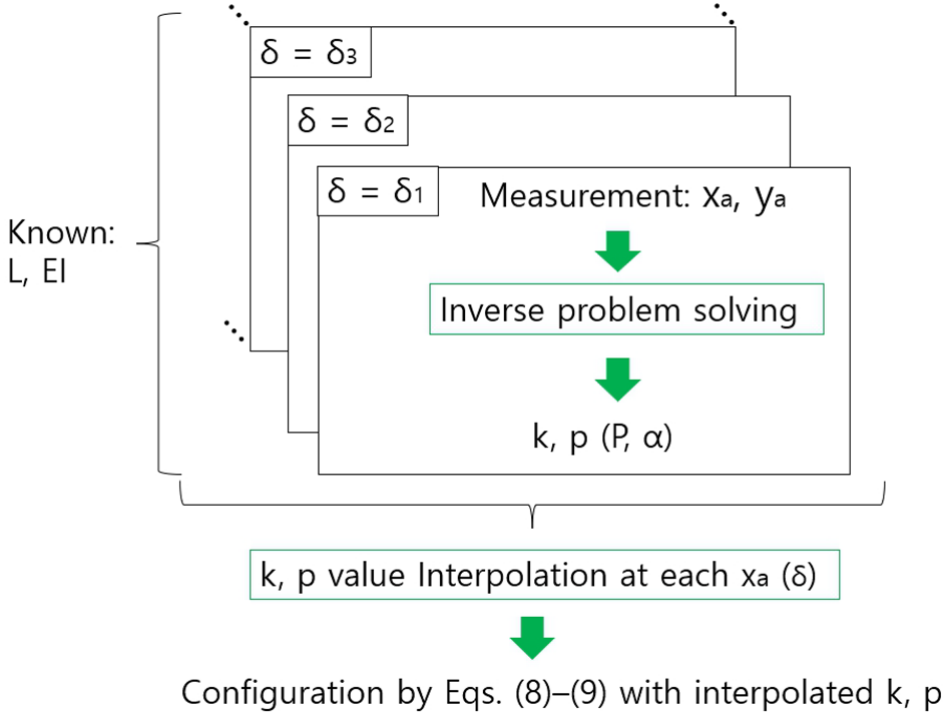
Overall sequence of a thin film strip buckling configuration determination.

With classical results of Eqs. (4–9), the buckling configuration including $x_{a}$ and $y_{a}$ of Fig. 2b can be calculated with *k*, *p*, and *EI* values, which is a forward problem (Input: *EI*, *k*, *p*, Output: configuration (y(x)) including $x_{a}$ and $y_{a}$)^9^. However, in the actual experiment using the mapping catheter prototype as presented in Fig. 2a, the known variables are $x_{a}$ and $y_{a}$ values but *k*, *p* values are unknown and must be determined to calculate the configuration of thin film strip with Eqs. (4–9), which is an inverse problem (Input: *EI*, *L*, $x_{a}$, and $y_{a}$, Output: *k*, *p* values). The proposed thin film buckling configuration determination method can solve the inverse problem at discrete *x*$_{a}$ and $y_{a}$ values and interpolate the solutions (*k* and *p*) at discrete $x_{a}$ values. The overall sequence is represented in Fig. 3.

In Fig. 3, the known parameters are *L* and *EI*. With a measurement value of $x_{a}$, $y_{a}$ at each $\delta$, the axial force *P* and initial bending angle $\alpha$ (*k* and *p*) can be calculated by the proposed method. These calculated discrete solutions of the inverse problem can be interpolated with respect to *x*$_{a}$ ($\delta$) and then, the configuration at any $x_{a}$($\delta$) can be calculated with the interpolated *k*, *p* value.

### Proposed inverse problem solution

#### Optimization solution by steepest descent method

The inverse problem in Fig. 3 can be converted to the optimization problem by applying the objective function, as shown in Eq. (10).

10 $$\begin{aligned} {f(p,k) = {(K(p) - k L)^2} + {(2p - k{{ y}_a})^2} + {(2E(p) - K(p) - k{{ x}_a})^2}} \end{aligned}$$

The first term in Eq. (10) is the quadratic form of the overall length difference between the analytic value and measurement value (*L*), which is derived by Eq. (4). The second and third term in Eq. (10) are also the quadratic forms of difference in the y$_{a}$ and x$_{a}$ values between the analytic values and measurement values (${y}_{a}$, ${x}_{a}$), which are from Eqs. (5)-(6). The optimization problem definition is given in Eq. (11).

11 $$\begin{aligned} \begin{array}{l} \mathop {\min }\limits _{p,k} f(p,k)\\ 0< p< 1,\mathrm{{ 0}}< k < 1000 \end{array} \end{aligned}$$

The range of *p* and *k* in Eq. (11) is determined by feasible *P* and $\alpha$ range, which are tracked by the thin film sensor in Fig. 2b. The steepest descent method^27,28^ was used first to determine the solution of Eq. (11) with the gradients with respect to *p* and *k* in Eqs. (12)-(13).

12 $$\begin{aligned}{} & {} {\frac{{\partial f(p,k)}}{{\partial p}} = 2(K(p) - k L)K'(p) + 4(2p - k{{ y}_a})}\nonumber \\{} & {}\,\,\,\,\,\,\,\,\,\,\,\,\,\,\,\,\,\,\,\,\,\,\,\quad + 2(2E(p) - K(p) - k{{ x}_a})(2E'(p) - K'(p))\nonumber \\{} & {}\,\,\,\,\,\,\,\,\,\,\,quad = 2K(p)K'(p) + 2(2E(p) - K(p))(2E'(p) - K'(p)) + 8p\nonumber \\{} & {}\,\,\,\,\,\,\,\,\,\,\,\,\,\,\,\,\,\,\,\,\,\,\,\,\,\quad - (2K'(p) L + 4{{ y}_a} + 2{{ x}_a}(2E'(p) - K'(p)))k \end{aligned}$$

13 $$\begin{aligned}{} & {} {\frac{{\partial f(p,k)}}{{\partial k}} = - 2(K(p) - k L) L - 2(2p - k{{ y}_a}){{ y}_a}}\nonumber \\{} & {}\,\,\,\,\,\,\,\,\,\,\,\,\,\,\,\,\,\,\,\,\,\,\quad - 2(2E(p) - K(p) - k{{ x}_a}){{ x}_a}\nonumber \\{} & {}\,\,\,\,\,\,\,\,\,\,\,\,\,\,\,\,\,\quad = - 2K(p) L - 2{{ x}_a}(2E(p) - K(p)) - 4p + 2({{ L}^2} + x_a^2 + y_a^2)k \end{aligned}$$

$E'(p)$ and $K'(p)$ in Eq. (12) are also in Eqs. (14)-(15).

14 $$\begin{aligned} K'(p)= & {} \frac{{E(p)}}{{p(1 - {p^2})}} - \frac{{K(p)}}{p} \end{aligned}$$

15 $$\begin{aligned} E'(p)= & {} \frac{{E(p) - K(p)}}{p} \end{aligned}$$

With Eqs. (10) and (12)-(15), the steepest descent method was temporarily tested using a screen protector of size 0.11$\times$4.0$\times$16.5 mm, made of polyethylene with Table 1 conditions. In Table 1, each parameter corresponds to the values in Fig. 2b. *x*$_{a}$ and *y*$_{a}$ in Table 1 are calculated by Eqs. (5) and (6) with *p* = 0.3826, *k* = 98.95. For the inverse problem setting, which calculates *k* and *p* values at *x*$_{a}$=14.04 and *y*$_{a}$=7.73 in Table 1, the initial *p* and *k* value are set to 0.5 and 500 because they are the half of the feasible range in Eq. (11). The screen protector strip size, *x*$_{a}$, and *y*$_{a}$ are decided to test the performance of the solver before applying it to the mapping catheter prototype in Fig. 2b, which is slightly larger than those of the mapping catheter prototype. The increment of *p* and *k* during the steepest descent method are set to 0.0001 and 0.1 and the terminal conditions are set to $\delta$p $\le$ 0.0001 and $\delta$k $\le$ 0.01 in total 10000 iterations. However, after the iteration number exceeds 100, the gradients of *k* and *p* keep oscillating, implying that the objective function in Eq. (10) has low value differences from alternating sign in the vicinity of optimal *k* and *p* value.

**Table 1:** Calculation condition—screen protector (thickness = 0.11 mm, width = 4.0 mm, Polyethylene material).

Variables	E (Pa)	I (10$^{9}$mm$^{2}$)	*P* (N)	$\alpha$ (deg)
Values	19607843	26.04	0.005	45.0

Variables	*k*	*p*	*L* (mm)	$x_{a}$ (mm)
Values	98.95	0.3826	16.50	14.04

Variables	$y_{a}$ (mm)			
Values	7.73			

**Figure 4:**
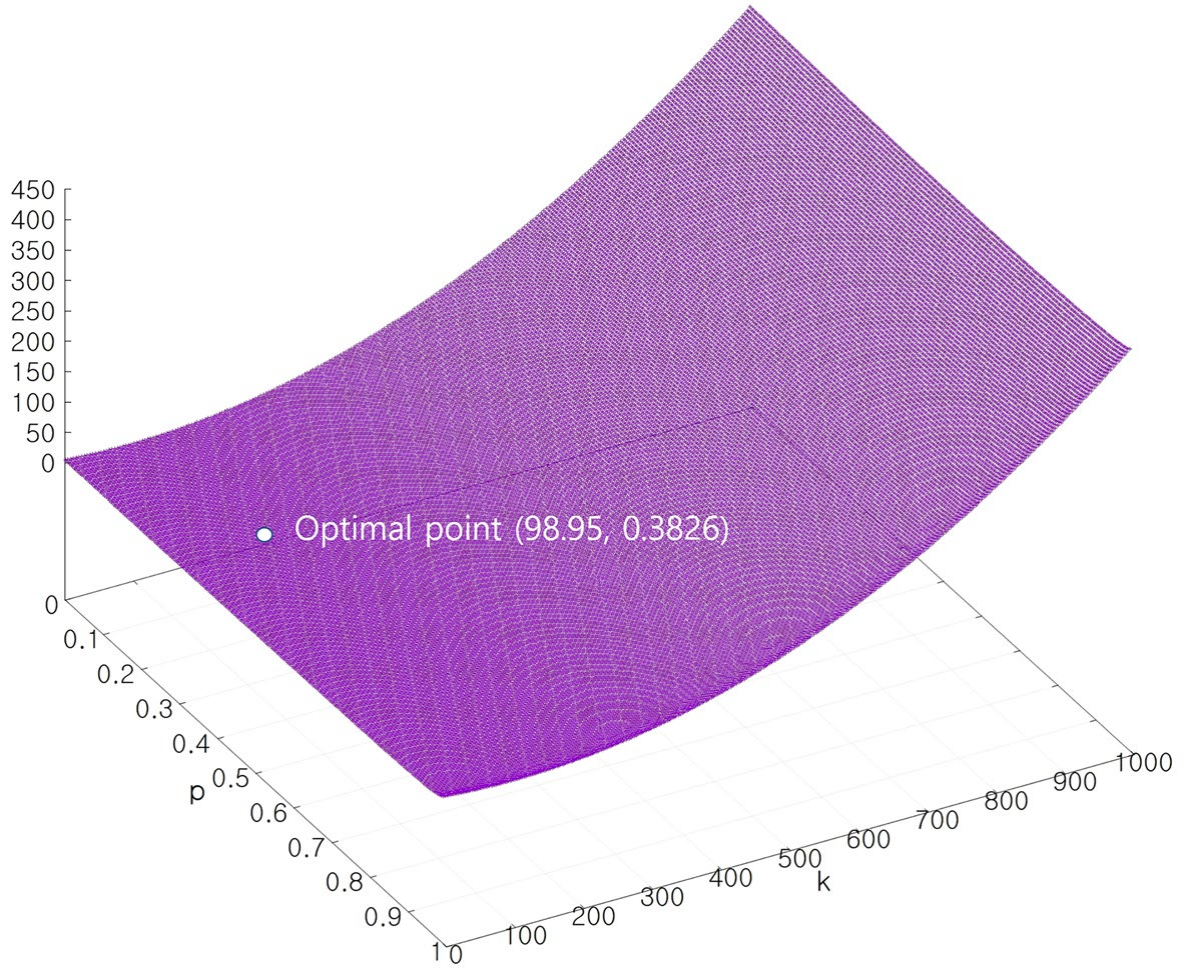
f(p,k) in Eq. (10) at *p*, *k* values.

*f(p,k)* value is plotted in Fig. 4 at each *p* and *k* values based on the result of oscillation. In Fig. 4, the *k* value ranges from 0 to 1000 and *p* value ranges from 0 to 1 as indicated in Eq. (11). The calculated optimal point is indicated by the white dot in Fig. 4. As described above, near the optimal point in Fig. 4, *f(p,k)* values have little value differences close to zero. Therefore, the steepest descent method is not a good candidate for solving the optimization (inverse) problem in this case.

#### Optimization solution by strip divide and conquer method

**Figure 5:**
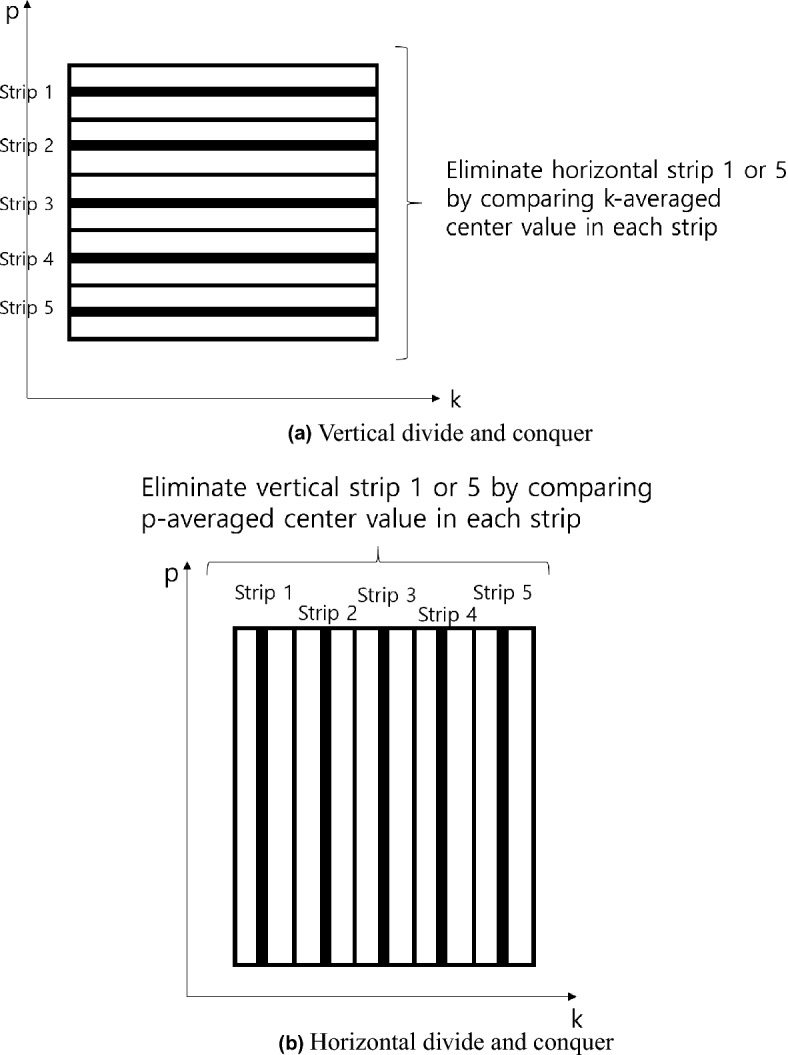
Schematics of strip divide and conquer method.

Based on the previous result and *f(p,k)* surface in Fig. 4, a strip divide and conquer method is proposed for solving the optimization problem in Eq. (11). The strip divide and conquer method is divided into a horizontal direction and vertical direction, which are represented in Figs. 5a,b.

In the vertical direction divide and conquer in Fig. 5a, *f(p,k)* surface in Fig. 4 is viewed from top and divided into fives equal sized strips vertically, numbered strip 1-5. The horizontal black lines in Fig 5a represent the center *p* value at each strip. The *f(p,k)* values of the black lines are averaged and compared to each other to eliminate the highest valued strip. In this manner, one of the five strips in Fig. 5a is eliminated. Similarly, one vertical strip can be eliminated by the horizontal divide and conquer, as shown Fig. 5b. By alternating the horizontal and vertical divide and conquer, the vertical and horizontal strips in Fig. 5 will gradually become smaller width strips, and eventually, the cross point of the narrow downed vertical and horizontal strips (*p* value in horizontal strip and k value in vertical strip in Fig. 5) is the optimal *p*, k value in the lowest *f(p,k)*.

The pseudo-code is shown in the Algorithm 1 below, where the vertical divide and conquer, and the horizontal divide and conquer are located at the upper and lower if statements, respectively. With the initial setting of variables, each if statement in the algorithm compares the current and previous *p(k)* values. If the absolute value of their difference exceeds $\delta$*p* or ($\delta$*k*), the comparison of each 5 stripes in Fig. 5a,b are performed by *compare_averaged_cost_function_value_at_p()* (*compare_averaged_cost_function_value_at_k()*). If the comparison is valid, the highest stripe is eliminated by *eliminate_p_range()* *(eliminate_k_range())* and update of *p(k)*. Then, the re-division of *p* range (*k* range) are sequentially performed by *update_p()* (*update_k()*) and *divide_p_range()* (*divide_k_range()*) functions. The if statement in the lower of Algorithm 1 is a breaking statement.

The result of Algorithm 1 with Table 1 condition is *k* = 98.954446 and *p* = 0.382684 (*P* = 0.00499999 N and $\alpha$ = 45.00007$^\circ$), which are almost equivalent to the answer *k* = 98.95522 and *p* = 0.382686 in Table 1. Through Algorithm 1, the *k* and *p* values (*P* and $\alpha$) can be calculated at various *x*$_{a}$ and $y_{a}$ measurement pairs at an increment of approximately 0.25 mm $x_{a}$ and each calculated *p*, *k* values is linearly interpolated with respect to *x*$_{a}$, which are presented in the next section.
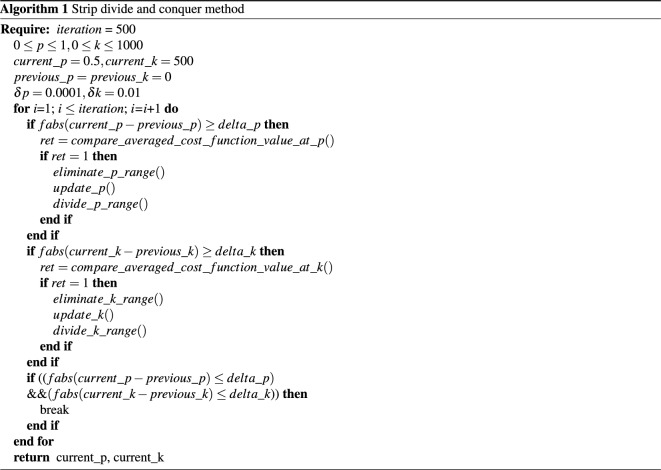


## Proposed method (algorithm 1), measurement, and FEM simulation results

### Screen protector strip

The screen protector strip in Table 1 is used for the preliminary test. Thickness and width of the strip is the same as that described in Table 1 (0.11 mm, 4.0 mm). However, *L* in Fig. 2a is set to 12.5 mm and *x*$_{a}$ is changed from 12.25 mm to 10.00 mm with an increment of the 0.25 mm (in 10 measurements) to make it deflect incrementally. In the measurements, the strip is placed on a small ruler to set the *x*$_{a}$ value at a specified length and each of its ends is fixed with tape. At each increment, the *y*$_{a}$ value is measured through graphical analysis. The FEM simulations are performed with ANSYS Workbench 22 R1^18^. The thin film is modeled as a thin plate with 600 mesh elements. To meet the predefined *x*$_{a}$ value, the boundary condition is simply supported at the left end and the force is applied to the right end of the film. Because the thin film has superelastic characteristics, plain buckling solver cannot simulate superelastic large deflection. Therefore, a coupling of structural and buckling solvers are used to make an initial buckling state, and the structural solver is used alone to deflect it according to the *x*$_{a}$ value after the initial buckling.

**Figure 6:**
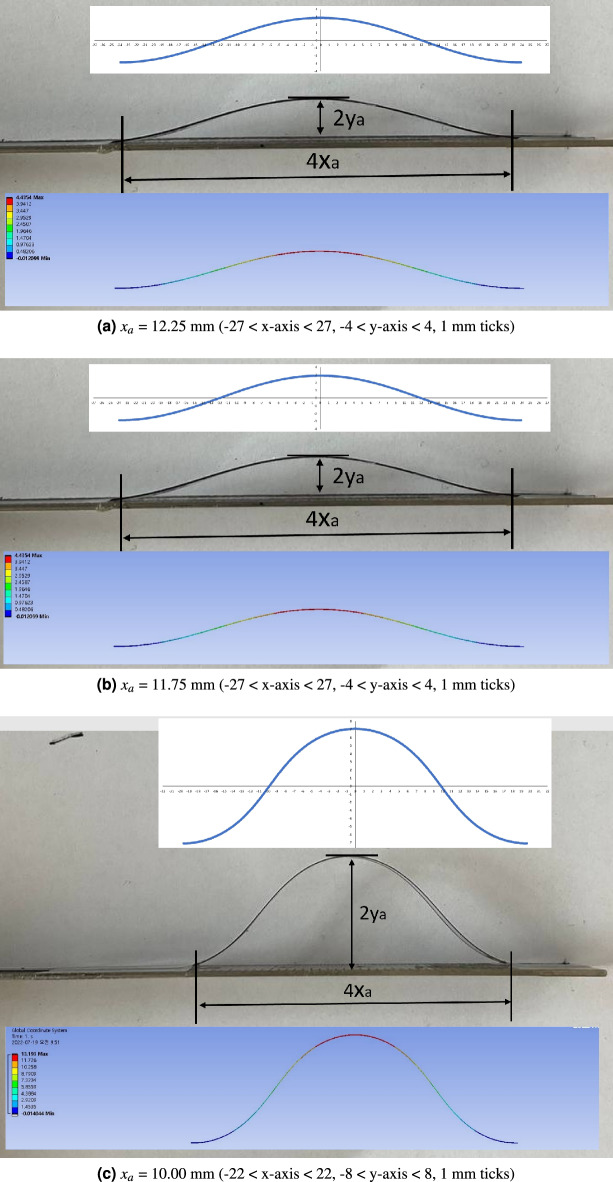
Screen protector strip deflection calculations, measurements and FEM simulations.

The calculation, measurement and FEM simulation result pictures are presented in Figs. 6a–c at *x*$_{a}$ = 12.25, 11.75, and 10.00 mm, respectively. As shown in Fig. 6a–c, the deflected configurations are almost identical in calculation, experiment and FEM simulation. With the experimentally measured *y*$_{a}$ values, the *k* and *p* values (*P* and $\alpha$ values) are calculated by Algorithm 1. With the calculated *k*, *p* values, *L*, *x*$_{a}$, and *y*$_{a}$ values are calculated by Eqs. (4–6) as presented in Calculation by Algorithm 1 column Table 2. Additionally, the experimental and FEM simulation results are presented in the 1st and 3rd column of Table 2.

**Table 2:** Screen protector strip results (*L* = 12.5 mm, 10 $\le$ ${x_{a}}$ $\le$ 12.25, 0.25 mm increment) [mm].

Experiment			Calculation by algorithm 1			Simulation by FEM		
*L*	*x*$_{a}$	*y*$_{a}$	*L*	*x*$_{a}$	*y*$_{a}$	*L*	*x*$_{a}$	*y*$_{a}$
12.50	12.25	2.56	12.53	12.21	2.55	12.50	12.25	2.32
12.50	12	3.57	12.57	11.93	3.54	12.50	12	3.22
12.50	11.75	4.30	12.59	11.66	4.26	12.50	11.75	3.89
12.50	11.5	4.95	12.63	11.38	4.89	12.50	11.5	4.45
12.50	11.25	5.28	12.60	11.16	5.22	12.50	11.25	4.93
12.50	11	5.57	12.57	10.93	5.53	12.50	11	5.36
12.50	10.75	6.32	12.68	10.59	6.21	12.50	10.75	5.74
12.50	10.5	6.86	12.76	10.29	6.69	12.50	10.5	6.09
12.50	10.25	6.96	12.69	10.10	6.82	12.50	10.25	6.40
12.50	10	7.68	12.86	9.74	7.42	12.50	10	6.70

**Figure 7:**
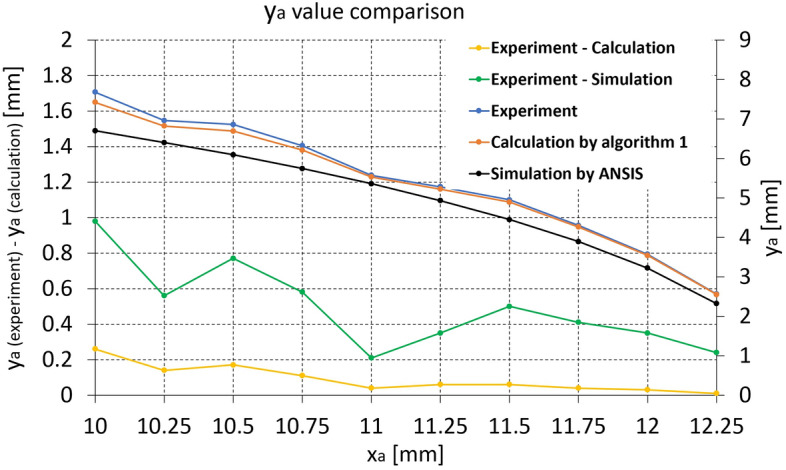
*y*$_{a}$ value comparison between experiment, calculation by Algorithm 1, and FEM simulation for the screen protector strip (Experiment—Calculation (yellow) and Experiment—Simulation (green) graphs are plotted against left axis).

The *L* and *x*$_{a}$ values in Experiment and Simulation by FEM columns in Table 2 are exactly the same due to the same boundary conditions. However, those in Calculation by Algorithm 1 columns in Table 2 are a little bit different compared to others because Algorithm 1 minimizes the objective function in Eq. (10), in which the three terms in Eq. (10) are not zero at the obtained k and p value.

Even though the optimization process cause very tiny difference in the *L* and *x*$_{a}$ values, the difference in *y*$_{a}$ value between the experiment and calculation (yellow line) is much smaller than that between the experiment and simulation (green line). In Fig. 7, the *y*$_{a}$ value decreases as *x*$_{a}$ value increases, which can also be observed in Fig. 6a–c.

### Mapping catheter sensor prototype

The mapping catheter sensor prototype, depicted in Fig. 1b has eight sensor strips, each of which has eight nodes to measure the ECG signal. The thickness and width of the strip are 0.13 mm and 0.9375 mm, respectively. *L* in Fig. 2b is set to 6.5 mm and *x*$_{a}$ is changed from 6.25 to 5.0 mm by an increment of 0.25 mm (total 5 measurements) to deflect it incrementally. The modulus of elasticity and Poisson’s ratio of the mapping catheter sensor prototype, which were measured through a tensile test by the local vendor, are 4.29 GPa and 0.34, respectively. The FEM simulations are performed as the same procedure as that of the screen protector strip.

By solving Eq. (11) with Algorithm 1, k and p values are obtained with the lumped material property, which is an error with respect to the true composite materials. However, this optimization process offsets the lumped material property error and calculates *k*, *p* values which best fit *L* and *x*$_{a}$, and measured *y*$_{a}$ values with the lumped material property. This is why *L* and *x*$_{a}$ values in Calculation by Algorithm 1 column in Table 3 are not exactly the same as those in Experiment and Simulation by AYSYS columns. Even though these offset makes *L* and *x*$_{a}$ values in exact, *y*$_{a}$ values at each *x*$_{a}$ and *L* values are superior compared to the following FEM results.

**Figure 8:**
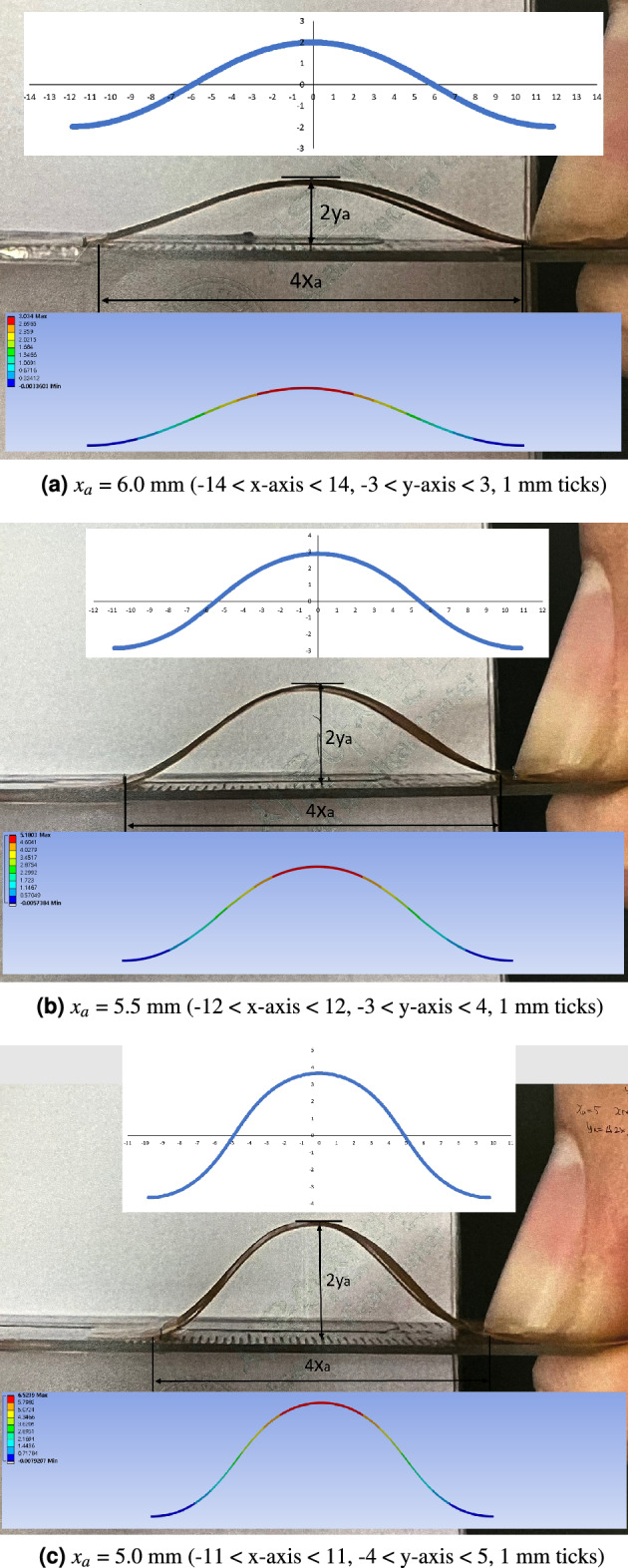
Mapping catheter sensor strip deflection calculation, measurements and FEM simulations.

The calculation, measurement and FEM simulation result pictures are presented in Figs. 8a–c at *x*$_{a}$ = 6.0, 5.5, and 5.0 mm, respectively. As shown in Fig. 8a–c, the deflection configurations of the experiment are close to that of the calculation and FEM simulation. The *L*, *x*$_{a}$, and *y*$_{a}$ values are calculated by Eqs. (4–6) to compare with experimental and FEM simulation results as presented in the Calculation by Algorithm 1 column Table 3.

**Table 3:** Mapping catheter sensor strip results (*L* = 6.25 mm, 5.0 $\le {x_{a}} \le$ 6.0, 0.25 mm increment) [mm].

Experiment			Calculation by Algorithm 1			Simulation by FEM 1		
*L*	*x*$_{a}$	*y*$_{a}$	*L*	*x*$_{a}$	*y*$_{a}$	*L*	*x*$_{a}$	*y*$_{a}$
6.25	6.0	2.01	6.32	5.92	1.98	6.25	6.0	1.64
6.25	5.75	2.56	6.33	5.67	2.52	6.25	5.75	2.21
6.25	5.5	2.90	6.31	5.44	2.86	6.25	5.5	2.72
6.25	5.25	3.52	6.41	5.12	3.42	6.25	5.25	3.08
6.25	5.0	3.75	6.39	4.89	3.64	6.25	5.0	3.39

**Figure 9:**
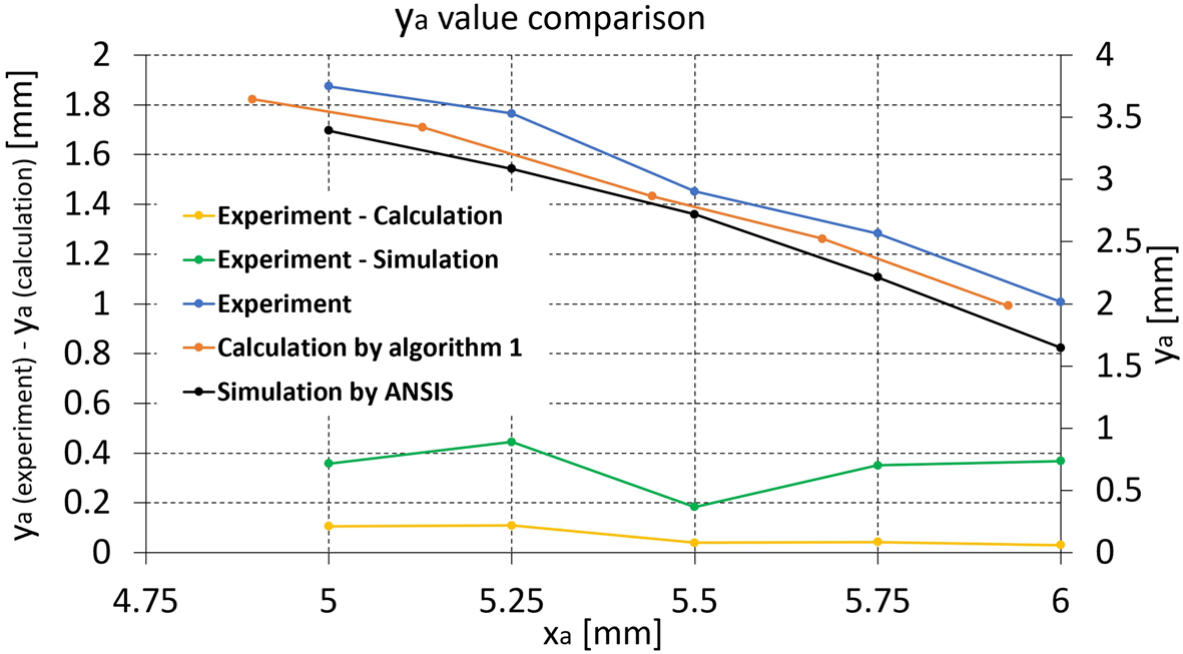
*y*$_{a}$ value comparison between experiment, calculation by Algorithm 1, and FEM simulation for the mapping catheter sensor prototype (Experiment—Calculation (yellow) and Experiment—Simulation (green) graphs are plotted against left axis).

*y*$_{a}$ value comparison between experiment, Algorithm 1, and FEM simulation for mapping catheter sensor strip is presented in Fig. 9. As shown in Fig. 9, the *y*$_{a}$ value difference between calculation and experiment (yellow line) is much smaller than that between the simulation and experiment (green line), which is similar to Fig. 7. The *y*$_{a}$ value difference between the experiment and calculation (yellow line) in Figs. 7 and 9 is lower than that between the experiment and simulation (green line) at all range of *x*$_{a}$ value, which means that the calculation by Algorithm 1 has superior than that of FEM simulation. The difference in *x*$_{a}$, *y*$_{a}$, *L* values between the calculation and experiment at each *x*$_{a}$ value are summarized in Table 4. As shown in Table 4, the maximum *L*, *x*$_{a}$, and *y*$_{a}$ difference between the calculation and experiment are 0.1643 mm, − 0.1221 mm, and − 0.1093 mm at Experiment *x*$_{a}$ = 5.25 mm, which are 2–3% at each overall measurement range. The RMSE of *L*, *x*$_{a}$, and *y*$_{a}$ in Table 4 are 0.1157, 0.08936, and 0.07405, respectively.

**Table 4:** *L*, *x*$_{a}$, and *y*$_{a}$ difference between the calculation and experiment for the mapping catheter sensor prototype [mm].

Experiment	Calculation—Experiment		
x$_{a}$	*L*	*x*$_{a}$	*y*$_{a}$
6	0.0784	− 0.0715	− 0.0303
5.75	0.0871	− 0.0755	− 0.0430
5.5	0.0692	− 0.0579	− 0.0394
5.25	0.1643	− 0.1221	− 0.1093
5.0	0.1467	− 0.1042	− 0.1056
RMSE	0.1157	0.08936	0.07405

## Conclusion

This study proposes an online thin film buckling configuration calculation using a parametric optimization for the mapping catheter prototype sensor application. To solve the inverse problem occurred in the practical use of the sensor, the divide and conquer type optimization algorithm is proposed, and it also overcome the fluctuation problem in gradient steepest descent algorithm. With the proposed framework, the lumped material properties errors and sensor strip defects are absorbed in the optimization procedure, which makes the configuration of the mapping catheter sensor strip prototype more accurate than those calculated from FEM software. Experiments and FEM simulations with a screen protector strip and the mapping catheter prototype sensor strip are performed and compared with the calculation result of the proposed method. The results confirmed that calculation results are closer to the experimental result than the FEM simulation result in that maximum difference of *L*, *x*$_{a}$, and *y*$_{a}$ values between the proposed method and the experiment are 0.1643 mm, − 0.1221 mm, and − 0.1093 mm, which are considered to have enough accuracy for flexible sensor applications of medical usage.

## Data Availability

All datasets used and/or analysed during the current study available from the corresponding author on reasonable request.
